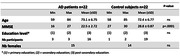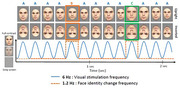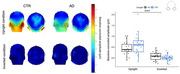# Preserved unfamiliar face identity discrimination ability in Alzheimer’s disease: electrophysiological evidence

**DOI:** 10.1002/alz.087243

**Published:** 2025-01-03

**Authors:** Justine David, Lisa Quenon, Bernard J Hanseeuw, Adrian Ivanoiu, Laurent Koessler, Bruno Rossion

**Affiliations:** ^1^ IMoPA, UMR 7365, CNRS‐Université de Lorraine, Nancy France; ^2^ Department of Neurology, Saint‐Luc University Hospital, Brussels Belgium; ^3^ Institute of Neuroscience, UCLouvain, Brussels Belgium; ^4^ Institute of Neuroscience ‐ UCLouvain, Brussels Belgium

## Abstract

**Background:**

While Alzheimer Disease (AD) patients’ difficulty to recognize face identity (Werheid & Clare, 2007) has been mainly attributed to episodic and semantic memory impairments, these patients can also show abnormal difficulties at matching of unfamiliar faces for their identity, suggesting impaired perceptual function (Lavallée et al., 2016). However, since this latter evidence is based on explicit behavioural measures, the difficulties of AD patients can be due to many factors (e.g., task understanding, stress, motivation, attention, decision making…).

**Methods:**

To obtain an objective and implicit measure of face identity discrimination ability we coupled electroencephalographic (EEG) recordings and fast periodic visual stimulation (FPVS). FPVS consists in the presentation of visual stimuli at a defined frequency that produces EEG responses at the exact same stimulated frequency. 22 diagnosed AD patients (CSF‐positive for AD, MMSE ≤27) and 22 healthy controls matched in sex and age (Fig.1) were tested with a well‐validated FPVS paradigm (>30 published studies) consisting in the presentation of a same identity face 6 times per second (base frequency: 6Hz) with variable other identities presented every fifth image (i.e., oddball frequency: 6/5 = 1.2Hz)(Fig.2). EEG responses at 1.2 Hz reflect an individual’s identity discrimination ability (e.g., Liu‐Shuang et al. 2014, *Neuropsychologia*; Rossion et al., 2020, *Eur. J. Neurosci*.).

**Results:**

At the 1.2 Hz oddball frequency and harmonics, a highly significant amplitude response was found at bilateral occipito‐temporal site for both groups (Fig.3; AD: 0.43 µV, p <.001; CTR: 0.58 µV, p <.001). Since this response becomes negligible when the same faces images are presented upside down (AD: 0.02 µV, p>.05; CTR:0.0009 µV, p >.05), the 1.2 Hz response reflect high‐level cognitive processes. ANOVA analyses revealed a significant effect of *Condition* (i.e., upright *vs*. inverted; p<.001). However, despite a lower response overall in AD patients (Fig.3), there was no significant effect of *Group* (p = .53) or interaction (p = .07).

**Conclusion:**

These results provide evidence with an implicit and objective neural measure that unfamiliar face identity discrimination ability is largely preserved in AD, suggesting that abnormal difficulties at matching pictures of unfamiliar faces for their identity in this population is due to other factors than visual discrimination.